# Development of Magnesium Alloy Stents with Layered Double Hydroxide Coating for Improved Corrosion Resistance and Biochemical Stability in AVF Applications

**DOI:** 10.3390/jfb17020076

**Published:** 2026-02-05

**Authors:** Chien-Hsing Wu, Fuh-Yu Chang, Chiung-Ju Lin, Ping-Tun Teng

**Affiliations:** 1 Department of Mechanical Engineering, National Taiwan University of Science and Technology, Taipei 106335, Taiwan; d10803005@mail.ntust.edu.tw; 2 SG Biomedical Co., Ltd., Hsinchu 30351, Taiwan

**Keywords:** autologous arteriovenous fistula, magnesium alloy stents, surface modification, layered double hydroxide, biochemical simulation

## Abstract

Background: Autologous arteriovenous fistula (AVF) is the most commonly used vascular access for end-stage renal disease patients. However, during the maturation process following AVF surgery, insufficient initial venous diameter often results in inadequate blood flow, leading to fistula maturation failure. Studies have indicated that implanting stents can enlarge the initial venous diameter and improve the success rate of AVF surgeries. However, stents made from metallic materials remain permanently in the body after implantation, posing risks such as in-stent restenosis. Methods: Our development and testing of magnesium alloy stents with a layered double hydroxide (LDH) coating to assist AVF maturation is presented in this paper. Firstly, AZ31 alloy was used as a benchmark to screen coating technologies, including anodizing, alkaline films, and LDH coatings. ZM21 tubes were then utilized to verify the transferability of optimized parameters across different substrates. Finally, the optimized coating was applied to ZM21 stents, followed by validation through in vitro degradation tests and biochemical simulations. Results: The results showed that LDH-coated AZ31 samples exhibited a 95% reduction in average corrosion rate compared to untreated samples. Additionally, the anion exchange property of the LDH layer effectively reduced the pH of the saline solution. Subsequently, LDH coatings were applied to ZM21 magnesium alloy stents, followed by in vitro degradation and biochemical simulation. Compared to untreated ZM21 stents, LDH-coated stents demonstrated a 94.9% reduction in average corrosion rate and significantly reduced the generation of soluble magnesium chloride, maintaining the solution pH below 8.0 and the Mg^2+^ concentration below 300 μg/mL. Conclusions: The results show LDH is the most effective corrosion-resistant coating and can control the degradation rate of magnesium alloy stents to enhance their support duration and biocompatibility.

## 1. Introduction

Hemodialysis is a type of renal replacement therapy. It uses a machine to extract blood, filter it through an artificial kidney, and remove toxins and excess water from the body, thereby substituting for the kidney’s detoxification function [1]. Before undergoing hemodialysis, it is necessary to establish a good vascular access site. Clinically, the commonly used vascular access methods are categorized into three types: autologous arteriovenous fistula (AVF), central venous catheter (CVC), and arteriovenous graft (AVG) [2]. The AVF procedure involves connecting an artery to a vein, utilizing the patient’s own blood vessels. Due to its high-quality vascular access, lower risk of occlusion, and fewer complications, AVF is considered the first choice [3]. However, achieving successful AVF maturation remains a significant challenge, with success rates ranging from 44% to 74% [3,4]. When AVF fails to mature or becomes occluded, percutaneous transluminal angioplasty (PTA) is used as an adjunct therapy. If PTA fails, metallic stent implantation may be considered [5].

Swinnen et al. [6] conducted a study on the use of metallic stents, which were placed at the arteriovenous anastomosis site via venous catheters. Results showed that 75% of patients achieved AVF maturation within six months, and 88% achieved maturation within 12 months. While metallic stents aid in maturation, they are not yet a definitive treatment due to potential long-term complications, such as restenosis and thrombosis [7]. Biodegradable vascular stents (BVSs), which metabolize over time in the body, overcome these limitations and eliminate the need for secondary surgeries [8].

Biodegradable metals, particularly magnesium-based alloys, have drawn significant attention due to their excellent biocompatibility and biodegradability. These materials have found applications in orthopedics and dentistry [9]. Studies on iron-based materials suggest good mechanical properties but slow degradation, often taking several years [10]. Pure zinc, while possessing an ideal corrosion rate, has a tensile strength of 120 MPa, far below the 300 MPa required for stent fabrication [11]. Pure magnesium degrades rapidly in aqueous environments, dissolving entirely within 1 to 3 months [12]. As a result, research has focused on magnesium alloys with tailored properties to balance strength and degradation rates [13,14].

Kandala et al. [15] developed an AZ31 magnesium alloy stent, which remained intact during a 28-day implantation period without life-threatening effects. However, concerns about potential toxicity from aluminum and heavy metals in some magnesium alloys, such as AZ31, have arisen. Manganese and zinc are relatively non-toxic alloying elements that also enhance corrosion resistance and mechanical properties [16]. ZM21 magnesium alloy, composed of magnesium, manganese, and zinc, has demonstrated a lower corrosion rate compared to AZ31 in vitro [17].

Magnesium alloy stents degrade rapidly, often being absorbed by the body too quickly to support vascular dilation adequately [18]. Surface treatments can improve stent performance by enhancing corrosion resistance [19]. For instance, Chen et al. [20] applied plasma electrolytic oxidation (PEO) to AZ31 magnesium alloy, achieving superior corrosion resistance at a specific current density. Additionally, anodization and hydrothermal synthesis methods have been used to create protective thin films, such as Mg(OH)_2_ and Mg-Al layered double hydroxide (LDH) thin films, on magnesium alloys [21,22,23,24]. These thin films improve corrosion resistance but are not sufficient for long-term protection against chloride ion-induced degradation.

LDH compounds, known for their unique structure, are widely researched for applications in supercapacitors, drug delivery, and corrosion protection [25]. Studies have shown the effectiveness of LDH coatings in improving the corrosion resistance of magnesium alloys [26,27,28,29,30]. However, the interaction between chloride ions and magnesium hydroxide can accelerate degradation, resulting in pH elevation and rapid magnesium ion release [31]. Elevated pH levels (>8.5) have been shown to significantly reduce cell viability [32].

This study aims to extend the degradation time of ZM21 magnesium alloy stents by using surface treatments to reduce the formation of soluble magnesium chloride. Initially, AZ31 magnesium alloy was employed as a well-documented and cost-effective benchmark for preliminary process screening, evaluating different surface treatments including anodization, the application of Mg(OH)_2_, and Mg-Al LDH thin films. Subsequently, ZM21 tubes were used to verify the transferability of the optimized LDH parameters across different substrates and to characterize film thickness and chemical composition under varying reaction times. Finally, the optimal treatment was applied to ZM21 stents, which were selected for their superior clinical safety as an aluminum-free material, thereby avoiding the potential neurotoxicity risks associated with AZ31. The goal is to achieve a post-degradation solution pH below 8.5 and magnesium ion concentrations below 300 μg/mL, making the stents suitable for AVF treatment applications. Biochemical simulation tests will validate the designed stents’ compatibility for biomedical use. ZM21 tubes will also be used to evaluate the thickness and chemical composition of thin films with different reaction times.

## 2. Materials and Methods

### 2.1. AVF Stent Implantation

The results from Swinnen et al. [6] and Aalami et al. [33] showed that by implanting stents at arteriovenous sites, AVF maturation success rate can be improved. However, due to the rapid degradation and cytotoxicity [34], magnesium alloy stents still possess issues that prevent them from being utilized fully. This study aims to develop a corrosion-resistant coating for magnesium alloy stents in order to improve the corrosion rate and lower toxicity of stents.

**Figure 1:**
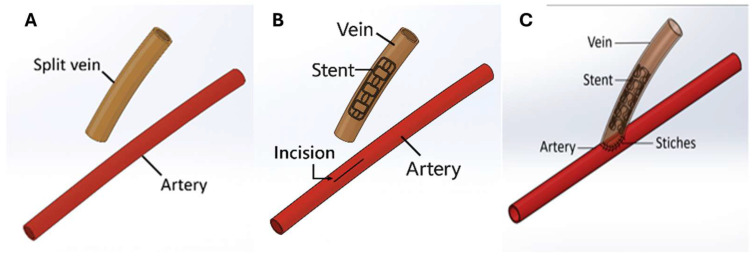
Implantation process of AVF. (**A**) Creation of a fan-shaped opening. (**B**) Stent placement. (**C**) Vein and artery connection.

The implantation process of an AVF stent is illustrated in Figure 1. First, the tissue surrounding the artery and vein was dissected. Hemostatic clamps were then used to clamp the vein and artery to stop bleeding. The vein was severed, and one side of the incision was cut axially to create a fan-shaped opening. Sutures were inserted into the incision for subsequent stitching (Figure 1A). After completing these steps, the compressed stent and balloon were inserted a few millimeters from the incision site via the vein. The balloon was inflated with water until the target pressure was reached, then deflated and removed to complete the stent placement (Figure 1B). An incision was made in the artery, and the sutures from the fan-shaped opening of the vein were threaded one by one around the arterial incision. This allows the fan-shaped vein incision to fully cover the arterial incision. The final bridging process was completed as shown in Figure 1C.

Due to magnesium-alloy-based stents being toxic and degrading rapidly after being exposed in blood, coatings on these stents can help alleviate the hazardous chemicals being released into blood vessels and also increase corrosion resistance. Therefore, various coatings, including alkaline thin films, anodized thin films and LDH thin films, were used to determine the best coating method.

### 2.2. Preparation of Magnesium Alloy Samples

**Figure 2:**
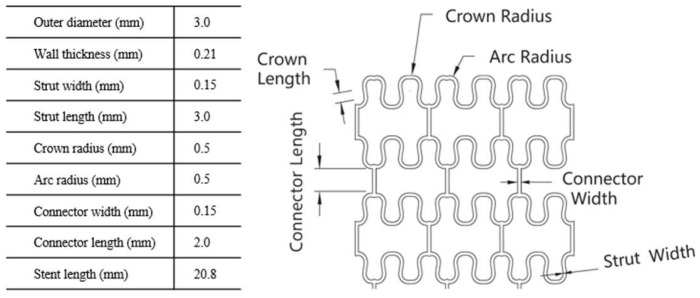
The stent pattern and specification.

The ZM21 stent for assisting AVF maturation was designed using Creo 3.0 and optimized with the assistance of ANSYS Workbench 2022 R2 (ANSYS Corp.; Pennsylvania; U.S.) finite element analysis software. The stent was composed of three fundamental structural units: Strut, Crown, and Connector. Each layer of the stent structure (Ring) consisted of four or six Crowns, and multiple Rings were connected by Connectors to form a complete stent. The stent designed in this study has an outer diameter of 3 mm, which can be compressed onto a 5Fr (1.7 mm) balloon catheter and expanded to an outer diameter of 5.4 mm, as shown in Figure 2. This design was based on the smallest recommended vein diameter of 2 mm for AVF, as well as the kidney disease outcomes quality initiative (KDOQI) guidelines, which recommend that a mature fistula should have a diameter of ≥6 mm [35]. However, some experts suggest that a mature fistula with a diameter of ≥5 mm is sufficient to begin dialysis [36]. Additionally, Feldman et al. [37] demonstrated that veins with a diameter greater than 5 mm have a 67% chance of maturing, while those with a diameter less than 5 mm have a maturation probability of less than 58%. Therefore, this study’s stent design aims for an outer diameter between 5 mm and 6 mm, with the expectation that the vein will mature successfully to a diameter exceeding 6 mm.

To determine the best coating method, AZ31 specimens were used first before experiments with ZM21 specimens. The AZ31 magnesium alloy plates were cut into samples measuring 10 mm × 10 mm × 4 mm. The cut samples were polished with #1200 silicon carbide sandpaper to remove oxide layers and contaminants, resulting in a smooth surface. The ZM21 magnesium alloy was processed into magnesium alloy stents using femtosecond laser machining. To determine thickness and chemical composition of the LDH thin films, ZM21 tubes were cut into dimensions of an outer diameter of 3 mm, inner diameter of 2.5 mm and length of 12 mm. Both the cut AZ31 and ZM21 specimens (Table 1) were ultrasonically cleaned in an acetone solution and subsequently dried in a hot air circulation oven. After fabrication, the stents were measured for dimensions using an optical microscope.

### 2.3. Fabrication of Thin Film Coatings

**Figure 3:**
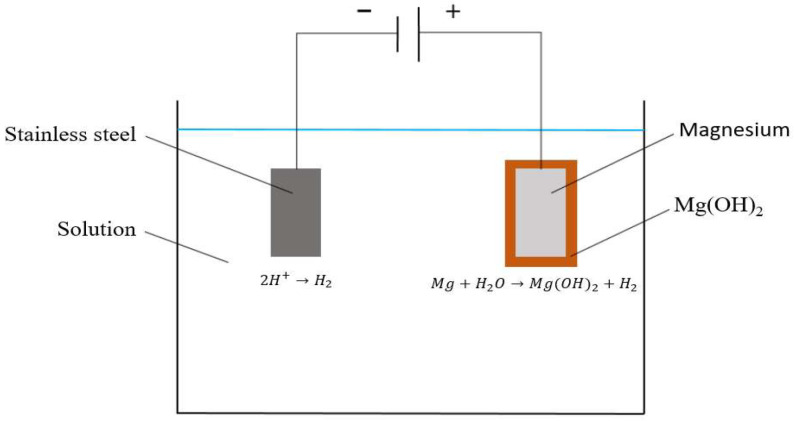
Schematic Diagram of the Fabrication Method for Anodized Coatings on Magnesium Alloy Samples.

The overall experimental design, including the specific labeling of all sample groups and their corresponding fabrication parameters such as voltage, hydrothermal temperature, and reaction time, is summarized in Table 2. To fabricate the anodizing thin film, the AZ31 magnesium alloy samples were connected to the anode (positive electrode) of a power source, while a stainless-steel electrode served as the cathode (negative electrode). These were placed in a 300 mL NaOH (1 M) solution, and a constant voltage of 3 V was applied. After reaction times of 10, 30, and 60 min, the samples were removed, rinsed with deionized water, and coated with Mg(OH)_2_ thin films (Figure 3). The samples were labeled as Anodizing-1, Anodizing-2, and Anodizing-3, respectively.

**Figure 4:**
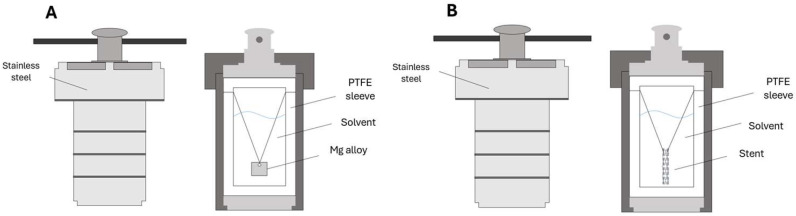
Fabrication Setup for (**A**) Surface Alkaline Film Synthesis. (**B**) Mg-Al LDH Synthesis.

To fabricate the alkaline thin film, the method used in this experiment was the hydrothermal synthesis process. The AZ31 magnesium alloy samples were placed in a PTFE-lined stainless-steel autoclave to create Mg(OH)_2_ thin films (Figure 4A). A mixture of 400 μL NaOH (10 M) solution and 50 mL deionized water was gently poured into the PTFE-lined autoclave containing the samples. The autoclave was then placed in a high-temperature oven maintained at 90 °C. The reaction times in the autoclave were 8, 12, 24, and 72 h. After the reaction, the samples were removed and rinsed with deionized water. These samples were labeled as Alkaline-1, Alkaline-2, Alkaline-3, and Alkaline-4, respectively.

To prepare Mg-Al LDH thin film (Figure 4B) for AZ31, the AZ31 magnesium alloy samples were placed in a PTFE-lined stainless-steel autoclave. A solution of 50 mL Al(NO_3_)_3_·9H_2_O (0.02 M) and 600 μL NaOH (10 M) was poured into the autoclave. The autoclave was placed in a high-temperature oven maintained at 120 °C. After 12 h, the samples were removed and rinsed with deionized water. For the ZM21 magnesium alloy stents, they were placed in a PTFE-lined stainless-steel autoclave. A solution of 50 mL Al(NO_3_)_3_·9H_2_O (0.02 M) and 600 μL NaOH (10 M) was poured into the autoclave. The autoclave was placed in a high-temperature oven maintained at 120 °C. After 12 h, the stents were removed and rinsed with deionized water. The AZ31 magnesium alloy sample was labeled as LDH-S.

A scanning electron microscope (SEM, Phenom XL, Thermo Fisher Scientific Inc.; Waltham, MA, USA) was used to observe the surface, cross-sectional morphology, and thin film thickness of the untreated AZ31 sample (Bare sample), AZ31-Mg(OH)_2_ samples, and Mg-Al LDH samples, LDH tubes and stents. Film thickness was determined from cross-sectional SEM images. For each specimen, five equally spaced points along the coating were selected, and the local thickness at each point was measured. The mean of these five measurements was reported as the coating thickness. The chemical composition and elemental distribution of the samples were evaluated using energy-dispersive X-ray spectroscopy (EDS, Phenom XL, Thermo Fisher Scientific Inc.; Waltham, MA, USA).

### 2.4. In Vitro Degradation Tests

**Figure 5:**
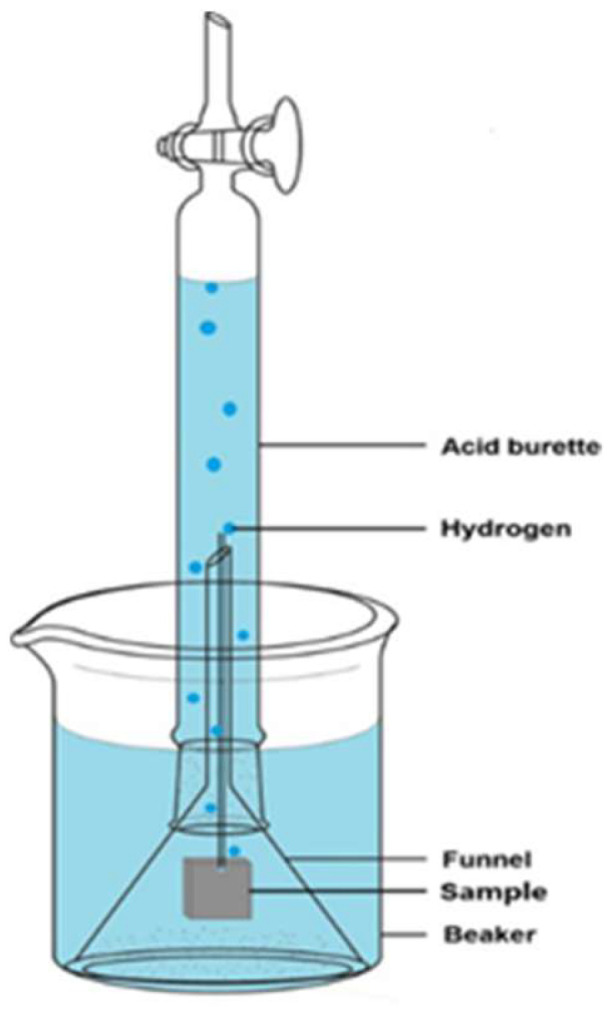
Hydrogen Evolution Experiment Model Framework.

The samples subjected to in vitro degradation testing were selected from the groups defined in Table 2 and categorized into two stages: (1) a screening stage using all AZ31 alloy groups (Bare, Anodizing 1 to 3, Alkaline 1 to 4, and LDH-S) to identify the optimal coating technology, and (2) a final validation stage using ZM21 magnesium alloy stents (Bare Stent and Stent LDH) to verify performance for the intended clinical application. A static in vitro degradation simulation experiment (Figure 5) was conducted to observe the degradation behavior of magnesium alloys in conditions mimicking in vivo placement. Changes in structure and corrosion rate over time were monitored. A pH meter (PH5011A, GOnDO Electronic Co., Ltd.; Taipei, Taiwan) was used to measure pH changes in the solution. The specimens were secured to a funnel with a string. The funnel was then placed upside down in a beaker. An acid burette was filled with Phosphate-buffered Saline (PBS) and placed over the funnel to achieve vacuum. As hydrogen gas is generated, it is collected at the top of the acid burette by displacing the PBS solution. The volume of evolved hydrogen was determined by daily recording the change in the liquid level on the acid burette’s graduated scale. PBS was used for the simulation, with a solution pH of 7.4, and the degradation process was conducted at 37 ± 1 °C. All the specimens were subjected to the same setup for the degradation test. Corrosion rates were measured, and results for the Bare sample, anodized samples, alkaline thin film samples, and the LDH-S sample were compared.

Hydrogen gas release was recorded daily for 7 consecutive days for each sample group. To evaluate the long-term corrosion resistance, the hydrogen evolution method was employed for more accurate corrosion rate measurements, providing more reliable data. During the reaction between magnesium and water, reaction products may remain on the magnesium alloy stents, Equation (1), leading to measurement errors. Therefore, the hydrogen evolution method was more precise than conventional weight measurement methods. The atomic mass of magnesium (24.305) was used in conjunction with Equations (2) and (3) to calculate magnesium mass loss. Based on the volume of hydrogen gas, the corrosion rate of the tested samples was calculated using Equation (4).

Mg + 2H_2_O → Mg(OH)_2_ (reaction product) + H_2_ (1)

Hydrogen molar number formula: P × V = n × R × T(2)

n: Moles of hydrogen gas; P: Standard atmospheric pressure; V: Volume of hydrogen gas released; R: Gas constant; T: Temperature during measurement.

(P × V(measured value))/(R × T) × 24.305 = Magnesium mass loss(3)

r = (P × V)/(R × T) × M/(A × t)(4)

r: Corrosion rate; P: Standard atmospheric pressure; V: Volume of hydrogen gas released; R: Gas constant; T: Temperature during measurement; M: Moles of hydrogen gas; A: Original surface area of the sample; t: Time of placement.

The corrosion rates of magnesium alloy stents with different thin film thicknesses vary, which allows for tailoring the degradation time to meet patient needs. ZM21 tubes were placed in an autoclave. A solution of 50 mL Al(NO_3_)_3_·9H_2_O (0.02 M) and 600 μL NaOH (10 M) was added to the autoclave, and the temperature was maintained at 120 °C. The reaction time for forming Mg-Al LDH thin films was varied, with three replicates per group. Tubular samples with a reaction time of 4 h were labeled as Tube LDH-1, while those with a 12 h reaction time were labeled as Tube LDH-2. The formation time, structure, and thickness of the Mg-Al LDH thin films were observed.

To evaluate the effects of increased solution concentration, the concentration was raised to 50 mL Al(NO_3_)_3_·9H_2_O (0.04 M) and 600 μL NaOH (20 M), while reducing the reaction time. The temperature was maintained at 120 °C. Tubular samples with a reaction time of 4 h were labeled as Tube LDH-3, while those with a 12 h reaction time were labeled as Tube LDH-4. The structure and thickness of the Mg-Al LDH thin films were evaluated to determine if results comparable to those obtained with a 0.02 M aluminum concentration could be achieved.

### 2.5. Simulation Tests for Extraction-Based Biochemical Evaluation of Mg-Al LDH ZM21-Coated Stents

This study simulated conditions under which ZM21 magnesium alloy stents were placed in extraction methods during biochemical simulation using a static in vitro degradation experiment. Stent LDH was placed in beakers and subjected to a water bath at 50 ± 2 °C. Hydrogen gas release was recorded daily for 3 consecutive days to observe structural degradation. Magnesium mass loss was calculated using Equations (2) and (3), and the magnesium ion concentration in the solution was determined using Equation (5):

Magnesium ion concentration = Magnesium ion mass/Solution volume(5)

The pH of the test solution during magnesium alloy degradation was monitored using a pen-type pH meter (PH5011A, EZDO, Taipei, Taiwan). At each predetermined time point, the retractable electrode was immersed in the solution, and the pH value was recorded after the reading stabilized.

## 3. Results

### 3.1. In Vitro Degradation of AZ31 Samples

**Figure 6:**
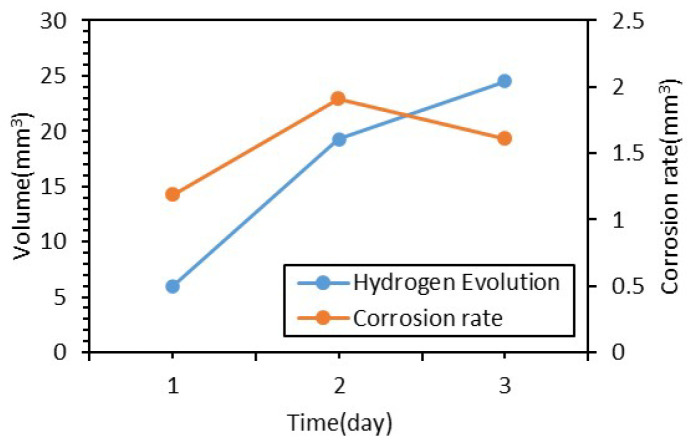
Hydrogen Evolution Curve and Corrosion Rate of AZ31 Bare Sample.

**Figure 7:**
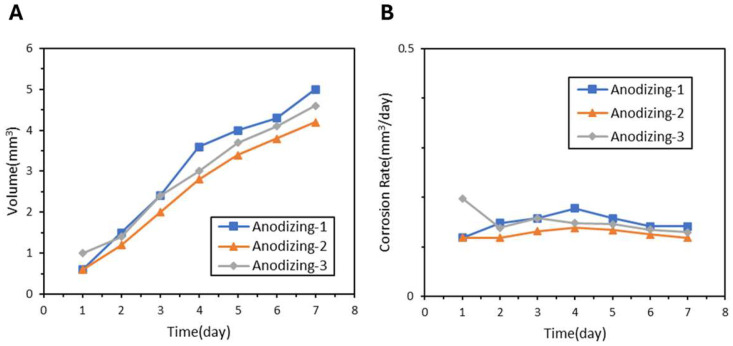
(**A**) Hydrogen Evolution Curve and (**B**) Corrosion Rate of Anodized Film Samples.

The in vitro degradation behavior of bare and anodized AZ31 magnesium alloy samples was assessed to evaluate their corrosion resistance in an aqueous environment containing chloride ions. The bare AZ31 samples exhibited rapid degradation as shown in Figure 6, where the samples released 24.5 mm^3^ of hydrogen over three days with a high average corrosion rate of 1.61 mm^3^ per day. Conversely, the anodized samples including Anodizing-1, Anodizing-2, and Anodizing-3 showed significantly enhanced resistance as illustrated in Figure 7A, which demonstrates that hydrogen evolution volumes were reduced to a range of 2.0 to 2.4 mm^3^. Figure 7B highlights that Anodizing-2 performed best among these samples with a corrosion rate of 0.11 mm^3^ per day.

**Figure 8:**
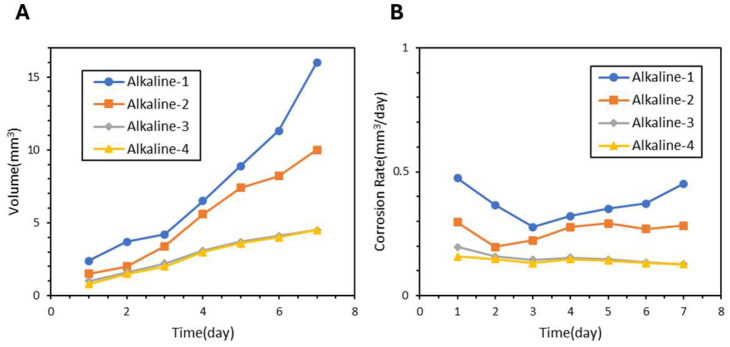
(**A**) Hydrogen Evolution Curve and (**B**) Corrosion Rate of Surface Alkaline Film Samples.

**Figure 9:**
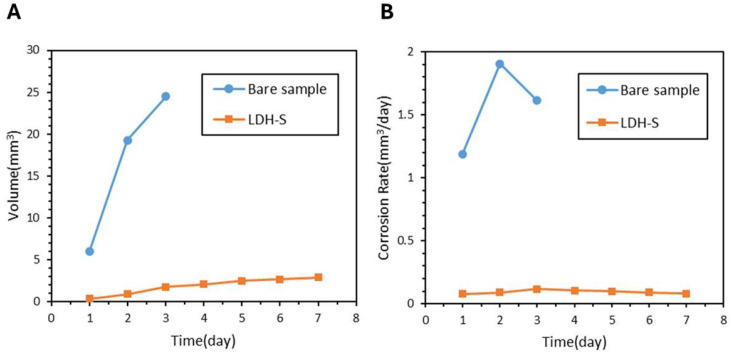
(**A**) Hydrogen Evolution Curve and (**B**) Corrosion Rate of Bare Sample and LDH-S Film Samples.

Alkaline-treated samples including Alkaline-1, Alkaline-2, Alkaline-3, and Alkaline-4 also showed improvements as demonstrated in Figure 8A. Figure 8B indicates that Alkaline-3 and Alkaline-4 reached the lowest rates of 0.12 mm^3^ per day each. The LDH-S sample demonstrated the most superior protection as seen in the hydrogen release of only 1.8 mm^3^ in Figure 9A and an average corrosion rate of 0.08 mm^3^ per day in Figure 9B. A comprehensive comparison of hydrogen evolution, corrosion rates, and pH values across all AZ31 coatings is summarized in Table 3. Notably, LDH-S maintained a solution pH of 8.11 after seven days, which was significantly lower than the 9.06 observed for bare samples.

### 3.2. Surface Characterization of AZ31 Coatings

**Figure 10:**
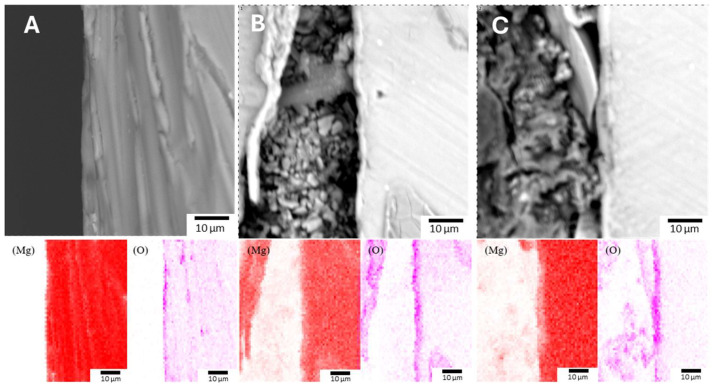
Cross-Sectional Morphology and Corresponding Mg and O Element Distribution. (**A**) Anodizing-2. (**B**) Alkaline-3. (**C**) LDH-S, with Darker Colors Indicating Higher Intensity.

EDS spectra confirmed the presence of magnesium, oxygen, and aluminum within these protective coatings, with the LDH-S sample showing the highest aluminum content at 2.71 at% as detailed in Table 4. It should be noted that while hydrogen is a fundamental constituent of the hydroxy compound layer, it could not be detected or identified in this study due to the inherent technical limitations of EDS in detecting light elements. Further insights into the coating structure were provided by cross-sectional imaging and elemental mapping. As illustrated in Figure 10, the elemental distribution maps for magnesium and oxygen showed varying intensities within the coating layers. These cross-section images indicated that the Anodizing-2 coating in Figure 10A possessed the greatest thickness at 8.62 μm. This was followed by the LDH-S coating shown in Figure 10C with a thickness of 2.85 μm, while the Alkaline-3 coating in Figure 10B was the thinnest at 2.66 μm. The dense surface layer observed in the cross-section of the LDH-S sample confirms the formation of a robust barrier that contributes to the enhanced structural integrity of the treated magnesium alloy

### 3.3. Evaluation of Mg-Al LDH Coated ZM21 Tubes

**Figure 11:**
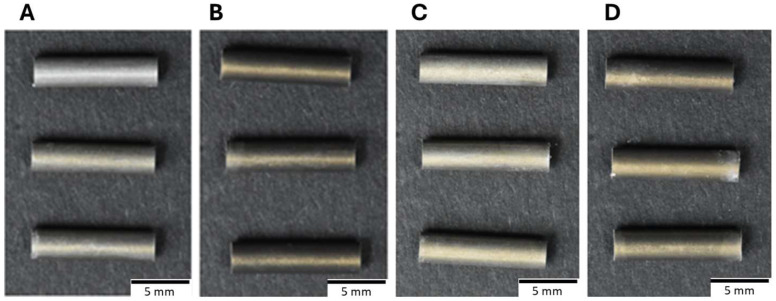
The appearance of (**A**) Tube LDH-1. (**B**) Tube LDH-2. (**C**) Tube LDH-3. (**D**) Tube LDH-4.

**Figure 12:**
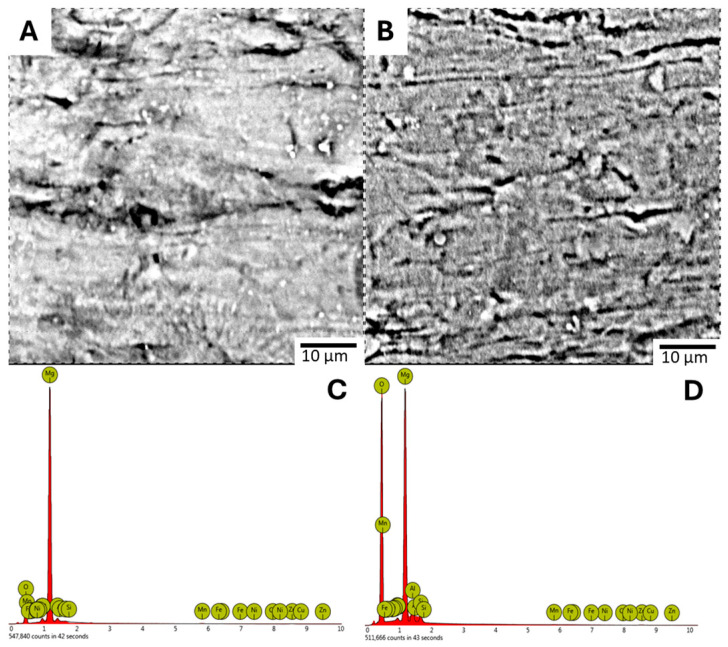
Surface Morphology of (**A**) Tube LDH-1. (**B**) Tube LDH-2. Corresponding EDS Spectra (**C**) Tube LDH-1. (**D**) Tube LDH-2.

**Figure 13:**
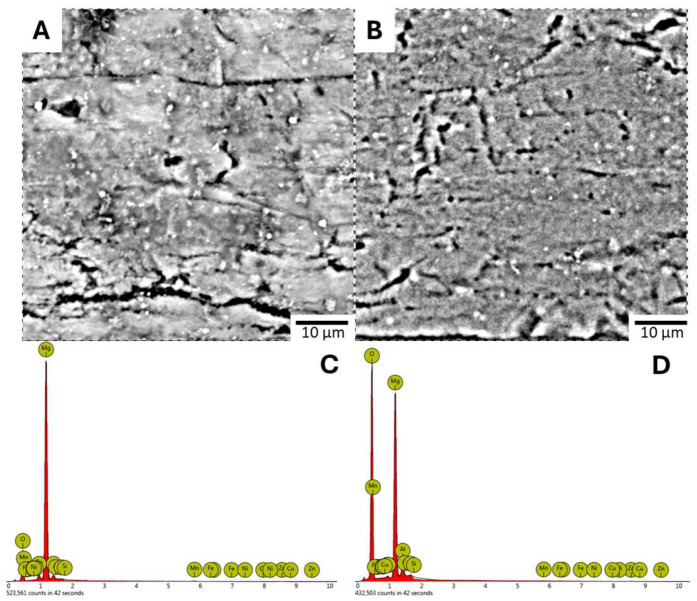
Surface Morphology of (**A**) Tube LDH-3 (**B**) Tube LDH-4; Corresponding EDS Spectra (**C**) Tube LDH-3, (**D**) Tube LDH-4.

The physical appearance of the coated ZM21 circular tubes is shown in Figure 11. For these tubes, the experimental results showed that increasing the solution molarity to 0.04 M while reducing the reaction time to 4 or 12 h achieved coating results that were comparable to or thicker than those obtained with 0.02 M concentrations as demonstrated in Figure 12 and Figure 13. Specifically, Figure 12A presents the surface morphology of Tube LDH-1 after a 4 h reaction, while Figure 12B displays the surface morphology of Tube LDH-2 following a 12 h reaction. The corresponding EDS spectra are illustrated in Figure 12C for Tube LDH-1 and Figure 12D for Tube LDH-2.

Elemental analysis revealed that Tube LDH-1 consisted of Magnesium at 77.66% and Oxygen at 22.72%, whereas for Tube LDH-2, the Oxygen content significantly increased over time to 61.80% while the Magnesium content decreased to 32.29%. The surface elemental compositions for the samples ranging from Tube LDH-1 through Tube LDH-4 are detailed in Table 5. Furthermore, Table 6 records that Tube LDH-4 reached the highest average thickness of 4.57 μm after a 12 h reaction.

### 3.4. Degradation and Biochemical Simulations of ZM21 Stents

**Figure 14:**
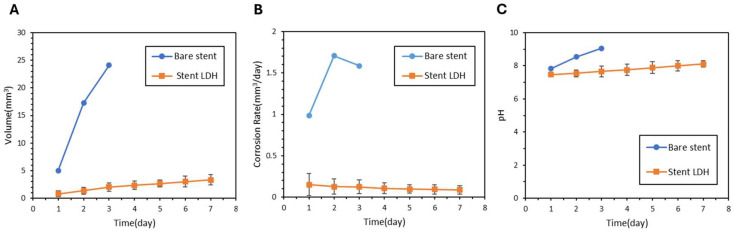
(**A**) Hydrogen Evolution Curve, (**B**) Corrosion Rate, and (**C**) pH Values of Bare Stent and Stent LDH.

**Figure 15:**
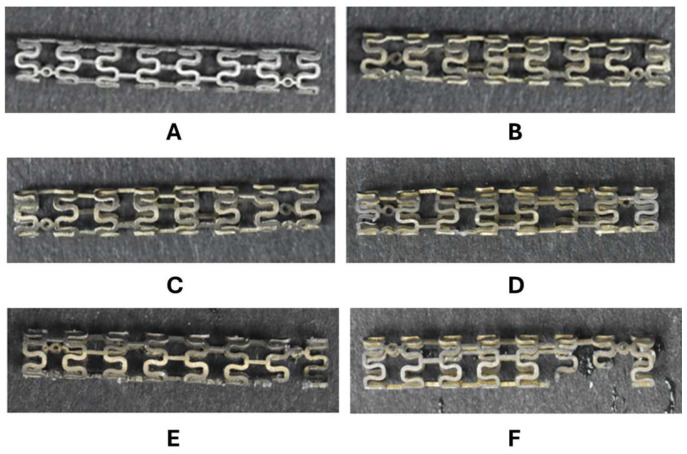
Mg-Al LDH ZM21 Film Stent Degradation Experiment: (**A**) Bare Stent. (**B**) Stent LDH. (**C**) Day 1. (**D**) Day 3. (**E**) Day 5. (**F**) Day 7.

The ZM21 Stent LDH demonstrated excellent performance throughout the degradation assessments. Figure 14A shows that the Stent LDH reached a hydrogen evolution of 2.03 mm^3^ after three days, which represents a 91.6% reduction compared to the 24.1 mm^3^ observed for bare stents. As illustrated in Figure 14B, the average corrosion rate of the LDH stent was 0.08 mm^3^ per day, reflecting a 94.9% reduction relative to the 1.58 mm^3^ per day of the bare stent. Visual monitoring of the degradation process from day 1 through day 7 is presented in Figure 15, showing that the Stent LDH maintained its structural integrity significantly longer than the bare stent.

**Figure 16:**
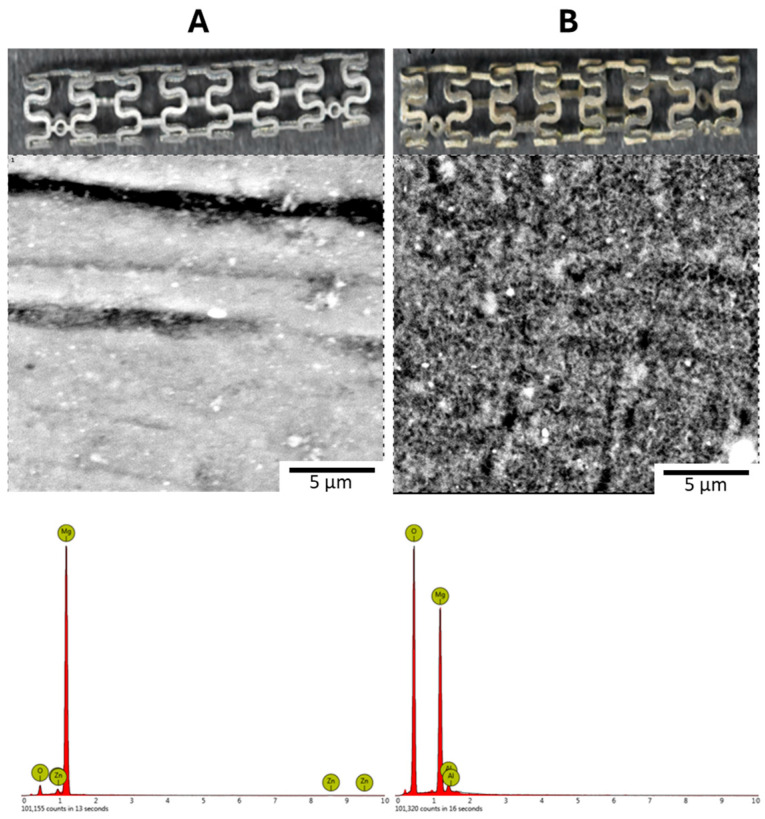
Surface Morphology and Corresponding EDS Spectra: (**A**) Bare Stent. (**B**) Stent LDH.

**Figure 17:**
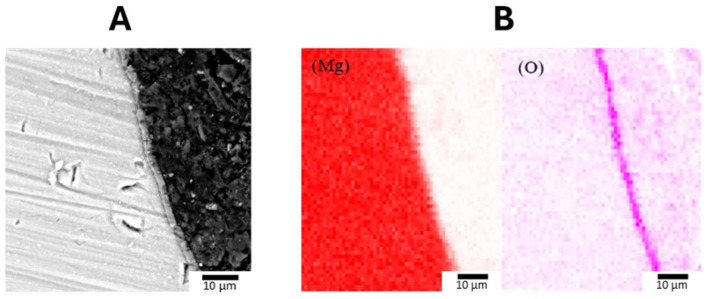
Characterization and Thickness of Stent LDH. (**A**) Cross-Sectional Morphology and (**B**) Corresponding Mg and O Element Distribution, with Darker Colors Indicating Higher Intensity.

**Figure 18:**
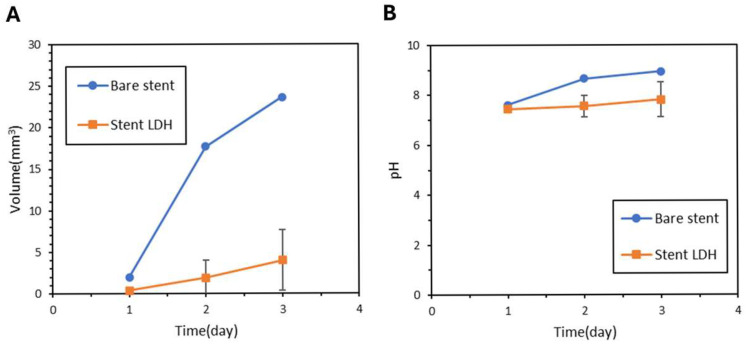
(**A**) Hydrogen Evolution Curve and (**B**) pH Values of Bare ZM21 stent.

Surface morphology and EDS analysis for the stents in Figure 16 and Table 7 confirmed the successful formation of the LDH coating layer. The cross-section morphology and the elemental distribution of Magnesium and Oxygen for the Stent LDH are presented in Figure 17, indicating an average film thickness of 2.71 μm. In the extraction-based biochemical simulations, the hydrogen evolution and pH trends are shown in Figure 18A and Figure 18B. The LDH-coated stents effectively maintained the solution pH around 7.8 and kept Magnesium ion concentrations between 64 and 300 μg/mL. As shown in Table 8, the samples for Stent LDH-a and Stent LDH-c maintained levels well below the cytotoxic threshold of 300 μg/mL.

## 4. Discussion

### 4.1. Summary of Key Findings

The development of the ZM21 magnesium alloy stent with a layered double hydroxide (LDH) coating addresses a critical clinical gap in assisting autologous arteriovenous fistula (AVF) maturation. While AVF is the gold standard for vascular access in hemodialysis patients, maturation failure remains a significant hurdle, with success rates ranging from only 44% to 74%. This failure is primarily attributed to insufficient initial venous diameter, which restricts blood flow. Although research by Swinnen et al. [6] demonstrated that implanting metallic stents could improve maturation success to 88% within 12 months, these permanent implants pose long-term risks such as in-stent restenosis and thrombosis. Biodegradable magnesium alloys offer a promising biocompatible alternative; however, their innate rapid degradation in chloride-rich environments often leads to premature structural failure, preventing sustained vascular support.

Our experimental results demonstrate that the Mg-Al LDH coating functions as a highly effective corrosion barrier, significantly outperforming traditional anodizing and alkaline surface treatments. Specifically, the LDH-coated ZM21 stents achieved a 94.9% reduction in average corrosion rate compared to untreated stents. The results highlight the excellent robustness and transferability of the optimized hydrothermal parameters (120 °C, 12 h) developed in this study. A comparative analysis across different substrates, such as the AZ31 sheet (LDH-S), the ZM21 tube (Tube LDH-2), and the ZM21 stent (Stent LDH), reveals highly consistent film thicknesses of 2.85 μm, 2.84 μm, and 2.71 μm, respectively. Furthermore, the functional performance remained remarkably stable, with the corrosion rate reduction being almost identical between the AZ31 sheet (95%) and the ZM21 stent (94.9%). This high degree of consistency in both physical dimensions and protective efficacy across different alloy compositions (AZ31 versus ZM21) and geometries (flat or tubular) demonstrates that the LDH coating process is highly controlled and can be reliably transferred from standard benchmark materials to complex, clinically relevant medical devices. This superior protection is derived from the dense structure of the LDH layer, which serves as a stable physical barrier and utilizes anion exchange properties to block aggressive chloride ions (Cl^-^) from reaching the magnesium substrate. This mechanism effectively inhibits the conversion of the Mg(OH)_2_ passivation layer into soluble magnesium chloride, which is a common cause of rapid degradation. Beyond corrosion resistance, the LDH coating acts as an active modulator of the biochemical interface by stabilizing the microenvironment during degradation. While bare magnesium stents cause rapid alkalization with pH levels exceeding 9.0, the LDH-coated stents maintained the solution pH around 7.8, which is well below the critical cytotoxic threshold of 8.5. Literature by Zhen et al. [32] indicates that pH values exceeding this threshold significantly reduce cell viability. Furthermore, magnesium ion concentrations were kept within safe physiological ranges (<300 μg/mL), ensuring excellent biocompatibility and reducing the risk of chemically induced intimal hyperplasia. These findings suggest that the developed stent can reasonably be expected to achieve the first and second clinical objectives when implanted in AVF veins; however, further animal studies are required to validate these outcomes in vivo

### 4.2. Study Limitations and Future Directions

Despite the significant advancements demonstrated in this study, several limitations must be considered to facilitate the clinical translation of these stents. A primary limitation is that the current evaluations relied on static in vitro degradation and biochemical simulations, which cannot fully replicate the complex, dynamic physiological environment of a living organism, such as pulsatile blood flow, variable shear stress, and long-term metabolic interactions. Furthermore, due to the destructive nature of the sampling process and the significant time required for fabrication, notably the alkaline treatment which can take up to 72 h per specimen, measurements were recorded as single data points across the extended timeline. While the absence of error bars limits localized statistical comparisons, the reliability of the findings is supported by the clear and consistent trends observed throughout the continuous experimental period. Moreover, the substantial magnitude of the protective effect offered by the LDH coating compared to the bare samples underscores the validity of the observed performance gap.

Additionally, technical challenges in the fabrication process were identified; specifically, the suspension method used during the hydrothermal synthesis created points of adhesion where the wire contacted the stent surface. This resulted in localized coating delamination upon wire removal in some samples, such as Stent LDH-b, leading to increased magnesium ion release at those specific sites. Furthermore, while structural integrity was maintained for the initial three days, significant corrosion damage was evident by the seventh day, suggesting the need for further refinement of the coating thickness to meet specific clinical duration requirements.

Future research will focus on addressing these issues through systematic process optimization and advanced physiological testing. To ensure process reproducibility and coating uniformity for potential large-scale production, new fixture designs will be developed to minimize contact areas during the film formation process. Furthermore, dynamic fluid simulation experiments integrated with quantitative mechanical evaluations, specifically radial stiffness, fatigue resistance, and ductility, will be implemented to assess the mechanical stability and degradation behavior of the stents under realistic blood flow conditions. Future work will also investigate the microstructural evolution of the LDH layer, incorporate in vitro biological evaluations including cytotoxicity, cell adhesion, and proliferation, and further optimize its long-term stability in physiological environments. Ultimately, comprehensive animal studies are required to track the long-term metabolic pathways of the LDH-coated ZM21 stents and to validate their actual performance in promoting vessel dilation and tissue repair in vivo. These advancements may eventually allow the application of Mg-Al LDH coatings to a broader range of biodegradable magnesium-based implants, such as orthopedic hardware or other cardiovascular devices.

## 5. Conclusions

This study systematically explored the effectiveness of Mg-Al LDH coatings in enhancing the corrosion resistance and biocompatibility of AZ31 and ZM21 magnesium alloys through a series of in vitro static degradation and biochemical simulations. For the AZ31 magnesium alloy, the application of different surface treatment methods revealed that Mg-Al LDH coating offered the most significant improvement in corrosion resistance. Specifically, LDH-S samples reduced the average corrosion rate by 95%, which was superior to the 93.2% and 92.5% reductions achieved by anodized and alkaline-coated samples respectively. The LDH film effectively minimized contact between the substrate and chloride ions in the solution, thereby reducing hydrogen evolution and maintaining lower pH levels.

For ZM21 magnesium alloy stents, the LDH-coated versions achieved a 91.6% reduction in hydrogen evolution and a 94.9% reduction in corrosion rate compared to untreated stents. These coated stents maintained a pH of 7.65 and exhibited structural integrity up to the third day of degradation, effectively addressing the rapid degradation issue observed in untreated ZM21 stents. These findings underscore the role of LDH coatings in prolonging the functional lifespan of stents under physiological conditions. Furthermore, biochemical simulation tests suggested the potential biocompatibility of the stents, as all coated versions maintained pH levels below 8.5 and magnesium ion concentrations below 300 μg/mL. The coatings effectively maintained the extraction medium environment within a physiological range, indicating a reduced risk of adverse biological reactions. In conclusion, this study demonstrates that Mg-Al LDH coatings significantly enhance corrosion resistance and ensure biocompatibility, making them a transformative approach for the development of magnesium-based AVF stents.

## Figures and Tables

**Table 1:** Elemental composition of AZ31 and ZM21.

	Mg (at%)	Al (at%)	Zn (at%)	Mn (at%)	Si (at%)	Cu (at%)	Ni (at%)	Fe (at%)
ZM21	97.94	N/A	1.12	0.30	0.10	N/A	0.22	0.19
AZ31	96.46	2.69	0.60	0.09	0.01	0.10	N/A	0.06

**Table 2:** Labeling of AZ31 Film Samples and ZM21 Film Stents.

Sample Labels	Magnesium Alloy	Thin Film Fabrication Method
Anodizing-1,2,3	AZ31	300 mL NaOH(1 M), Constant voltage 3 V, 10 min, 30 min, 60 min
Alkaline-1,2,3,4	AZ31	400 μL NaOH (10 M) + 50 mL deionized water, 90 °C, 8 h, 12 h, 24 h, 72 h
LDH-S	AZ31	50 mL Al(NO_3_)_3_·9H_2_O (0.02 M) + 600 μL NaOH(10 M), 120 °C, 12 h
Stent LDH	ZM21 stent	50 mL Al(NO_3_)_3_·9H_2_O (0.02 M) + 600 μL NaOH(10 M), 120 °C, 12 h
Tube LDH-1	ZM21 circular tube	50 mL Al(NO_3_)_3_·9H_2_O (0.02 M) + 600 μL NaOH(10 M), 120 °C, 4 h
Tube LDH-2	ZM21 circular tube	50 mL Al(NO_3_)_3_·9H_2_O (0.02 M) + 600 μL NaOH(10 M), 120 °C, 12 h
Tube LDH-3	ZM21 circular tube	50 mL Al(NO_3_)_3_·9H_2_O (0.04 M) + 1200 μL NaOH(20 M), 120 °C, 4 h
Tube LDH-4	ZM21 circular tube	50 mL Al(NO_3_)_3_·9H_2_O (0.04 M) + 1200 μL NaOH(20 M), 120 °C, 12 h

**Table 3:** Comparison of Experimental Results for Anodizing-2, Alkaline-3, and LDH-S.

Day 7	Hydrogen Evolution (mm^3^)	Corrosion Rate (mm^3^/Day)	pH
Anodizing-2	4.2	0.11	8.52
Alkaline-3	4.5	0.12	8.56
LDH-S	2.9	0.08	8.11

**Table 4:** Corresponding Elemental Composition.

	Mg (at%)	O (at%)	Al (at%)
Bare sample	96.46	-	2.69
Anodizing-2	88.64	9.21	2.14
Alkaline-3	33.58	63.87	1.58
LDH-S	35.33	61.27	2.71

**Table 5:** Corresponding Elemental Composition.

	Mg (at%)	O (at%)	Al (at%)
Tube LDH-1	77.66	22.72	2.41
Tube LDH-2	32.29	61.80	3.94
Tube LDH-3	67.23	27.36	2.80
Tube LDH-4	30.52	65.11	3.42

**Table 6:** Comparison of Mg-Al LDH Film Thickness Measurement Results.

	Average (*n* = 3)
Tube LDH-1	0.86 ± 0.03 μm
Tube LDH-2	2.84 ± 0.08 μm
Tube LDH-3	0.89 ± 0.04 μm
Tube LDH-4	4.57 ± 0.03 μm

**Table 7:** EDS Analysis of Elemental Composition on the Surface of ZM21 Samples with and without LDH Coatings.

	Mg (at%)	O (at%)	Al (at%)
Bare stent	87.14	12.09	-
Stent LDH	29.32	68.77	1.91

**Table 8:** Comparison of Mg-Al LDH ZM21 Coating Stent Extraction Method Simulation Results.

Day 3	Original Weight (g)	Hydrogen Evolution (mm^3^)	Mg^2+^ Mass Loss (g)	Mg^2+^ Concentration (μg/mL)	pH
Bare stent	0.0242	24.1	0.0216	864	8.84
Stent LDH-a	0.0241	1.8	0.0016	64	7.73
Stent LDH-b	0.0237	8.2	0.0075	300	7.94
Stent LDH-c	0.0239	2.0	0.0018	72	7.75

## Data Availability

The original contributions presented in the study are included in the article; further inquiries can be directed to the corresponding author.
